# Disproportionate Rates of COVID-19 Among Black Canadian Communities: Lessons from a Cross-Sectional Study in the First Year of the Pandemic

**DOI:** 10.1007/s40615-023-01903-z

**Published:** 2024-01-22

**Authors:** Upton D. Allen, Michelle Barton, Julia Upton, Annette Bailey, Aaron Campigotto, Mariana Abdulnoor, Jean-Philippe Julien, Jonathan Gubbay, Niranjan Kissoon, Alice Litosh, Maria-Rosa La Neve, Peter Wong, Andrew Allen, Renee Bailey, Walter Byrne, Ranjeeta Jagoowani, Chantal Phillips, Manuela Merreles-Pulcini, Alicia Polack, Cheryl Prescod, Arjumand Siddiqi, Alexander Summers, Kimberly Thompson, Sylvanus Thompson, Carl James, for the seroMARK Research Group, Pamela Appelt, Mark Awuku, Paul Bailey, Janet Collins, Liben Gebremikael, Jenny Gumb, Tesfai Mengesha, Adaoma Patterson, Cheryl Prescod, Noelle Richardson, Sylvanus Thompson, Nicole Welch

**Affiliations:** 1 Division of Infectious Diseases, Department of Paediatrics, The Hospital for Sick Children, 555 University Avenue, Toronto, ON M5G 1X8 Canada; 2 Division of Infectious Diseases, Department of Paediatrics, London Health Sciences Centre, London, ON Canada; 3 Division of Allergy and Immunology, Department of Paediatrics, The Hospital for Sick Children, Toronto, ON Canada; 4 Toronto Metropolitan University, Toronto, Ontario Canada; 5 Division of Microbiology, Department of Paediatric Laboratory Medicine, The Hospital for Sick Children, Toronto, ON Canada; 6 Research Institute, The Hospital for Sick Children, Toronto, ON Canada; 7 Public Health Ontario, Toronto, Canada; 8 Department of Paediatrics, University of British Columbia, Vancouver, Canada; 9 Department of Paediatrics, The Hospital for Sick Children, Toronto, ON Canada; 10 Black Creek Community Health Centre, Toronto, Ontario Canada; 11 Dalla Lana School of Public Health, University of Toronto, Toronto, ON Canada; 12 Middlesex-London Health Unit, London, Ontario Canada; 13 Toronto Public Health Unit, Toronto, Ontario Canada; 14 York University, Toronto, ON Canada

**Keywords:** COVID-19, Black residents, Polymerase chain reaction

## Abstract

**Background:**

Racialized communities, including Black Canadians, have disproportionately higher COVID-19 cases. We examined the extent to which SARS-CoV-2 infection has affected the Black Canadian community and the factors associated with the infection.

**Methods:**

We conducted a cross-sectional survey in an area of Ontario (northwest Toronto/Peel Region) with a high proportion of Black residents along with 2 areas that have lower proportions of Black residents (Oakville and London, Ontario). SARS-CoV-2 IgG antibodies were determined using the EUROIMMUN assay. The study was conducted between August 15, 2020, and December 15, 2020.

**Results:**

Among 387 evaluable subjects, the majority, 273 (70.5%), were enrolled from northwest Toronto and adjoining suburban areas of Peel, Ontario. The seropositivity values for Oakville and London were comparable (3.3% (2/60; 95% CI 0.4–11.5) and 3.9% (2/51; 95% CI 0.5–13.5), respectively). Relative to these areas, the seropositivity was higher for the northwest Toronto/Peel area at 12.1% (33/273), relative risk (RR) 3.35 (1.22–9.25). Persons 19 years of age or less had the highest seropositivity (10/50; 20.0%, 95% CI 10.3–33.7%), RR 2.27 (1.23–3.59). There was a trend for an interaction effect between race and location of residence as this relates to the relative risk of seropositivity.

**Interpretation:**

During the early phases of the pandemic, the seropositivity within a COVID-19 high-prevalence zone was threefold greater than lower prevalence areas of Ontario. Black individuals were among those with the highest seroprevalence of SARS-CoV-2.

## Introduction

Severe acute respiratory syndrome coronavirus 2 (SARS-CoV-2) infections and the illness that results (COVID-19) have resulted in a pandemic with profound consequences [1]. COVID-19 cases have been disproportionately concentrated within racialized communities. Among these groups, Black people have been profoundly affected in the UK, the USA and Canada [2–4].

COVID-19 cases are typically diagnosed by polymerase chain reaction (PCR) testing that is performed on nasopharyngeal swabs [5–7], and PCR testing protocols have largely been conducted only in those with symptoms of COVID-19. Given the significant rates of asymptomatic infections, PCR testing likely underestimates the prevalence of infection within communities, unless widespread testing of symptomatic and asymptomatic person is done. Serologic testing enables a more complete assessment of the extent to which SARS-CoV-2 infection and associated outcomes have affected communities [8, 9]. Furthermore, this enables risk factors that are associated with infection to be elucidated, including the presence of medical comorbidities and social determinants of health, including those that define material deprivation indices [10].

This study examined the use of serologic testing for SARS-CoV-2 infection in a community-based sample from Ontario, Canada, to determine the full extent to which SARS-CoV-2 has penetrated into the Black community, which cannot be captured by current PCR testing protocols. One of our goals was to establish feasibility of enrolling large number of Black Canadians, a population that has had a long history of mistrust of the health care system. We sought to determine the extent of community participation and the seroprevalence of SARS-CoV-2 antibodies in the study population. We compared the seropositivity rates among persons in lower socioeconomic settings with a large proportion of Black residents compared with more affluent settings with lower percentages of Black residents. We also examined risk factors that are associated with seropositivity for SARS-CoV-2 infection, including but not limited to the presence of medical comorbidities and the markers of the social determinants of health [11].

Our study was a prelude to a more extensive study of the seroprevalence of SARS-CoV-2 among Canadian communities with the largest proportions of Black residents. A goal was to get a truer sense of the actual prevalence of COVID-19 infection in these communities, in contrast to relying solely on PCR test results from symptomatic patients, prior to the start of COVID-19 vaccination campaigns. Furthermore, the study was intended to provide valuable data from the Black community, a group that is often not well represented in research projects, including community-based projects.

## Methods

### Study Design

We conducted a prospective cross-sectional study from August 15, 2020, to December 15, 2020, with a planned follow-up phase of up to 18 months after enrollment. The first phase of COVID-19 vaccination started on December 15, 2020 [12]. Thus, persons in this study would not have received vaccines at the time of blood sampling. In this report, we have addressed the cross-sectional component of the study.

### Study Setting

We conducted a community-based study in three regions of Ontario during a period prior to the start of the provincial COVID-19 vaccination efforts. The primary area of focus had a higher proportion of Black residents than most areas of Canada. In Toronto, this was an area that had a higher proportion of Black residents (> 15–30%) compared to the provincial proportion for the province of Ontario, Canada (4.7%) [13]. Thus, we focused on the northwest quadrant of the Greater Toronto Area (GTA) and nearby suburban area of Peel, Ontario. We also aimed to enroll participants from areas that covered a spectrum of the socioeconomic levels across the province given that socioeconomic status might be associated with the likelihood of exposure to SARS-CoV-2. Thus, additional regions of focus included the suburban city of Oakville, Ontario, and London, Ontario. These areas have predominantly White residents in contrast to the northwest quadrant of the GTA. The northwest quadrant of the GTA and Peel are known to be COVID-19 hot spots (high case counts) based on the COVID-19 cases reported, whereas Oakville and London, Ontario, are regarded as lower incidence areas.

Northwest quadrant of the Greater Toronto Area and Peel Region: The northwest quadrant of the Greater Toronto Area is an expansive region in southern Ontario, Canada. The northwest quadrant lies to the north of the Toronto city centre, a major economic and cultural hub. It is situated to the east of Hamilton, a city known for its industrial and waterfront districts. Within the northwest quadrant, the area where the study was focussed has 29% Black residents. Adjoining this area is the suburban area of Peel Region with 15.3 Black residents [14]. The median household income of the northwest quadrant of the GTA is Can $66,000 [15].

Oakville, Ontario, is a suburban town located to the southwest of Toronto along the shores of Lake Ontario. It is recognized for its picturesque waterfront and affluence relative to neighbouring communities. The median household income is Can $128,000, which is well above the Ontario provincial figure of $91,000 [16, 17]. It is positioned to the south of Toronto, east of Hamilton, Ontario. The proportion of Black residents in this area is 2.9% [16].

London, Ontario, is a city in southwestern Ontario, known for its educational institutions. It is located to the west of Toronto and approximately 120 miles northwest of Niagara Falls, which is on the border of Ontario and New York State. The proportion of Black residents in London is 3.5% [18]. The median household income is Can $76,500 [18].

### Study Population and Sample

Participants consisted of Black Canadians and non-Black Canadians (White, South Asian, East Asian, Middle East, Hispanic, Other) residing in the areas where the study was conducted. We recruited participants using different promotional approaches that included social media, a study website, conventional media (radio interviews) and promotion using flyers and information sheets that were distributed to potential participants within the communities. Flyers were distributed at community social events, churches and health centres. The promotional material encouraged the participation of Black Canadians to obtain enough of these participants for meaningful analyses and generalizability. Persons were eligible for inclusion if they were greater than 2 years of age. We excluded persons who had symptomatic COVID-19 at the time of enrollment to ensure the safety of the research team. Participants were invited to participate at the study enrollment sites and written informed consent was obtained. In addition, we obtained assent for age-appropriate children. Participants received gift certificates as approved by the Research Ethics Board and no financial compensation was provided.

### Study Procedure

Participants were administered a questionnaire that included items relating to clinical, epidemiologic, demographic and socioeconomic variables. Clinical data included age, gender, chronic medical conditions and medications that could potentially affect the immune responses to SARS-CoV-2. Epidemiologic, socio-economic, demographic data and ethno-racial information were collected. The race/ethnicity categories used were adapted from those used by Statistic Canada [19]. Types of occupation were categorized as frontline versus non-frontline, where the former include but are not limited to persons working as teachers, health care workers, protective service workers (e.g. police, fire, and emergency medical services), personal support workers, taxi drivers and cashiers. Each participant provided a 10-cc venous blood sample which was stored at 4°C followed by processing within 7 days. Separated sera were stored at −20°C or colder prior to the performance of the immunoassay.

We established a community advisory group (CAG) to facilitate community engagement [20]. The group included members from a cross-section of the Black Canadian community primarily and included non-Black Canadians working or living within the Black community. Regular meetings were held between the research team and the CAG who were all unpaid volunteers. Besides the CAG, the research team worked closely with leaders from the communities where enrollment occurred.

### Primary Outcome Measurement

Testing was performed at the Hospital for Sick Children, Toronto. SARS-CoV-2 IgG antibodies were determined using the EUROIMMUN assay which is a Health Canada-approved enzyme-linked immunoassay (EUROMMUN, Lubeck, Germany) [21]. This is a semi-quantitative assay that targets recombinant S1 protein of SARS-CoV-2. The interpretation of results was based on the signal to cutoff ratios of < 0.8 being reported as negative, ≥ 0.8 to < 1.1 as borderline and ≥ 1.1 as positive [21]. The sensitivity of the assay is reported to be 94.4% with a specificity of 99.6% among persons who are > 10 days after the onset of symptoms [21].

### Research Ethics

Research Ethics approval was obtained from Clinical Trials Ontario (CTO; Project ID CTO 3274), the Hospital for Sick Children (3274-CIA-Aug/2020-42457) and the London Health Sciences Centre (reference 3274-CIA-Nov/2020-45486).

### Statistical Analyses

We estimated that a sample size of 300 participants would produce greater than a 95% confidence interval equal to the sample proportion plus or minus 0.02 when the estimated seropositivity proportion is 0.03. Data were descriptively summarized, and proportions compared using chi-square of Fisher’s exact test, as appropriate. Seropositivity estimates were expressed as proportions with 95% confidence interval (Binomial exact estimates). Continuous variables were compared using Student’s *T* test or a non-parametric procedure, as appropriate. Risk ratios (relative risk) were determined for different SARS-CoV-2 exposure categories. The relationship between seropositivity and different variables is examined using univariate analyses and a multiple logistic regression approach including the interaction between variables such as race and location of residence. For the multivariable analyses, we included variables that were primarily aligned with our main objectives (location of residence and race) and others achieving the lowest *P* values on univariate analyses. 95% confidence intervals were provided where appropriate. There were very few missing data (1%) and we proceeded with analysis as if we had a complete dataset.

## Results

### Descriptive Characteristics (Table 1)

We recruited 388 participants from across the study sites; however, one participant withdrew from the study without providing a reason**.** Most of the participants we enrolled were from the northwest quadrant of the Greater Toronto Area and adjoining areas of Peel Ontario, where 70.5% (273/387) participants were enrolled. The proportions of participants enrolled from Oakville and London were 15.5% (60/387) and 13.2% (51/387)), respectively. Most participants were at or above the age of 19 years (87.1%), with 57.9% in the 31–65 years age group. Participants less than 19 years accounted for 12.9% of enrollees. The breakdown according to race indicated that we were successful in recruiting Black Canadians: Black Canadians 65.9% (255/387), non-Black Canadian 33.9% (131/387; race/ethnicity unknown for 1 participant) and among non-Black persons, those identifying as White accounted for 15.3% (59/387), representing the second largest racial group of participants enrolled.

**Table 1:** Descriptive characteristics of study participants

	*N*	Percentages
Participants		
Total	387	100
London	51	13.2
Oakville	60	15.5
Peel	67	17.3
GTA Northwest	206	53.2
Other/unknown	3	0.8
Gender		
Males	162	41.9
Females	221	57.1
Prefer not to answer	1	0.26
Other	3	0.80
Race/ethnicity		
Black Canadian	255	65.9
Non-Black Canadian	131	33.9
Unknown	1	0.26
White	59	15.3
South Asian	20	5.2
East Asian	17	4.4
Hispanic	24	6.2
Middle East	4	1.03
Other	7	1.8
Age groups (yrs)		
< 19	50	12.9
19–30	62	16.0
31–65	224	57.9
> 65	45	11.6
Not available	6	1.6

Table 2 shows a comparison between the study population and general population groups in three areas of Ontario, Canada. We had deliberately focused on enhancing participation of Black Canadians. The study group had proportionately more women relative to men compared with the general population. The study population had a median age of 45 years compared with the three general population groups with median ages of 38, 39.5 and 41.6 years, respectively.

**Table 2:** Comparison of characteristics of study sample to general population

Statistic	Study sample	Northwest Quadrant, Toronto*	Toronto	Ontario
Median age (yrs)	45	38	39.5	41.6
Male to female ratio	1:1.4	1:1.1	1:1.1	1:1
Families with children at home	51.6%	34%	39%	39.6%
Median income	-	66,500	84,000	91,000
Most prevalent income categories in study group	< $25,000 (20.7%) $25,000 to < $49,999 (17.3%) $50,000–$74,999 (14.2%)	n/a	n/a	n/a
Ethnoracial groups				
Black participants	65.9%	28.8%	9.6%	5.5%
White	15.3%	26.0%	40.3%	65.7%
South Asian	5.2%	9.3%	14.0%	10.8%
East Asian	4.4%	2.9%	12.7%	6.8%
Hispanic	6.2%	9.1%	3.3%	1.8%
Other	3.1%	13.3%	8%	5.6%

*For this comparison, we have focussed on the general population area where the largest number of study participants reside, the city of Toronto and the province of Ontario, Canada

To examine the extent to which participants might have been motivated to enter our study to determine their antibody status following previous COVID-19 infection, we evaluated a subset of participants. Thus, among a subset of participants for whom there were complete data on COVID-19 exposures and diagnosis, 14.2% (27/190) indicated that they felt that they had COVID-19 respiratory symptoms prior to serological testing. Among these persons, 4.2% (8/190) tested positive for COVID-19 antibodies. From among 150 subjects who provided complete data on prior PCR testing, two participants who were previously PCR-positive for COVID-19 were also seropositive in our study.

### Seropositivity

Table 3 shows that the seropositivity for the overall study population was 9.6% (95% CI 7.1–13.3). Seropositivity values for London and Oakville were comparable (3.9% (2/51; 95% CI 0.5–13.5) and 3.3% (2/60; 95% CI 0.4–11.5), respectively). Using, the Oakville/London estimate as the reference, the seropositivity for the GTA northwest region was 3.5-fold greater (RR 3.5, 95% CI 1.3–9.8), seropositivity 12.3% (95% CI 8.1–17.6). Black persons in the Northwest quadrant of Toronto had a seropositivity of 14.4% (95% CI 9.4–19.5). The seropositivity rate for Black participants residing in London/Oakville was 1.5% (95% CI 0.04–8.0). Among 59 participants who identified as White (50.9% of whom resided in Oakville/London), the seropositivity was 3.4% (95% CI 0.41–11.7). As shown in Table 3, the highest seropositivity values were observed in the < 19 years age group 22.0% (95% CI 11.5–36.0). Pairwise comparisons showed a statistically significant difference between the seropositivity among the latter age group compared with the 31–65 years age groups (*P* < 0.01) with no significant difference when compared with the 19–30 age group *P* = .09).

**Table 3:** Summary of seropositivity rates among major categories of subjects

Categories	Seropositivity values (%)	95% CI
Overall	37/387 (9.6)	6.8–13.0
London	2/51 (3.9)	0.5–13.5
Oakville	2/60 (3.3)	0.4–11.5
Peel	7/67 (10.5)	4.3–20.4
GTA Northwest	26/206 (12.6)	8.4–17.9
Other	0/3 (0)	0–0.71
Black Canadian	28/255 (11.0)	7.4–15.5
Non-Black Canadian	9/131 (6.9)	3.2–12.6
White	2/59 (3.4)	0.41–11.7
South Asian	3/20 (15.0)	3.2–37.9
East Asian	2/17 (11.8)	1.5–36.4
Middle East	0/4 (0)	0–0.60
Hispanic	2/24 (8.3)	1.0–27.0
Other*	0/7 (0)	0–0.41
Age groups (yrs)		
< 19	10/50 (20.0)	10.3–33.7
19–30	5/62 (8.1)	2.7–17.8
31–65	16/224 (7.1)	4.1–11.3
> 65	6/45 (13.3)	5.1–26.8
Not available	0/6 (0)	0–0.50
Income categories (persons > 19 yrs)		
< $25,000	6/80 (7.5)	2.8–15.6-
$25,000 to < $49,999	4/67 (6.0)	1.7–14.6
$50,000–$74,999	6/55 (10.9)	4.1–22.3
$75,000–$99,999	5/35 (14.3)	4.8–30.3
$100,000–$149,999	2/27 (7.4)	0.9–24.3
$150,000 or more	0/20 (0)	0–0.17
No income or prefer not to answer	0/28 (0)	0–0.12
Unknown	4/25 (16.0)	4.5–36.1

*Ethnoracial information not provided for 1 additional participant

### Factors Associated with Seropositivity

We found that three postal code neighbourhoods in Toronto and Peel accounted from greater than 50% of all seropositive cases (M3N, L6A and L4T) and were the areas with > 10% of participants being seropositive. Univariate analysis indicated that being a resident of the GTA/Peel area was more than three times likely to as associated with seropositivity when compared with residence in London/Oakville (RR 3.43 (95% CI 1.30–8.03). Using White as the reference, there was a statistical trend for Black persons to be more likely to be seropositive compared with White persons (RR 3.24 95% CI 0.79–13.22; *P* = .07); similar estimates for the other groups were as follows: South Asian RR 4.4 (95% CI .80–24.6; *P* = 0.1); East Asian 3.5 (.53–22.84; *P* = .21); and Hispanics 2.46 (.37–16.46; *P* = .58). The seropositivity estimates among Black versus South Asian, East Asian and Hispanics were not statistically significantly different. Being a frontline worker compared to a non-frontline worker was associated with a greater risk of seropositivity (RR 2.69 (95% CI 1.0–7.23), *P* = 0.04. There were no significant differences in seropositivity among males versus females; RR 1.08 (95% CI 0.58–2.01), *P* = 0.82, while having a link with schools (teachers or students) showed a trend to be associated with seropositivity RR 1.85 (95% CI 0.96–3.57), *P* = 0.06.

### Multivariable Model

Table 4 shows univariate analyses and variables that were included in multivariable analyses. We examined the association between seropositivity and age, race and location of residence and the interaction between race and location of residence (model 1) and age, race and location of residence (model 2). Persons < 19 years were 2.5 times more likely to be seropositive compared with their older counterparts, RR 2.5 (95% CI 1.26–4.78). Residence in the GTA/Peel area was associated with a greater risk of seropositivity, RR 3.5 (95% CI 1.26–9.53). With respect to the interaction between race and location of residence, for Black Canadians compared with non-Black, the adjusted RR for seropositivity for residence in the GTA/Peel region was 1.70 (95% CI 0.72–4.04), and in contrast for the Oakville/London location, the RR was 0.17 (95% CI 0.02-1.59); therefore, the RR increased approximately tenfold for Black participants residing in the GTA/Peel Region; however, this increase was not statistically significant (*P* = 0.06; RR = 9.75 (CI 9.3 to 105.25).

**Table 4:** Evaluation of variables potentially associated with seropositivity

Variables	*N*	Seropositive (*N* = 37)	Seronegative (*N* = 350)	Unadjusted RR (95% CI)	Univariate *P*-value	Multivariable regression RR (95% CI)	
						Model 1	Model 2
GTA/Peel Other (reference)	273 113	33 4	240 109	3.41 (1.24–9.42)	0.01	3.29 (0.99–10.83) *P* = 0.94	3.5 (1.26–9.53) *P* = 0.02
Race: Black Non-Black (reference)	255 131	28 9	227 122	1.59 (0.77–3.22)	0.19	0.57 (0.17–1.89) *P* = 0.14	1.3 (0.63–2.7) *P*=0.48
Age: <=19 y >19 y (reference)	50 131	10 27	40 304	2.27 (1.23–3.59)	0.01	2.48 (1.28–4.78) *P* < 0.01	2.5 (1.26–4.78) *P* < 0.01
Interaction location & race	NA	N/A	N/A	N/A	0.06	9.75 (9.3–105.25) *P* = 0.06	

## Discussion

In the USA, during the early phases of the pandemic, SARS-CoV-2 seroprevalence varied across states between 5 and 25% [22–24]. In Canada, the seroprevalence for the general population was estimated to be less than 5% based on data derived from blood donors [25]. Data are limited on the SARS-CoV-2 seroprevalence for Black communities in North America. Our report provides insight into such estimates in Ontario, Canada, during the second wave of COVID-19. Overall, the results show that the seroprevalence detected among persons in a COVID-19 hot zone was several times greater than estimates derived from blood donors across Canada [25]. This confirms that certain communities were been disproportionately affected by the virus.

This study directs its primary focus towards the Black community within a North American context, acknowledging the alarming disparities in the impact of COVID-19. However, it is essential to underscore that this focus on the Black community does not diminish the recognition that other racialized groups have also experienced significant disparities in the face of the pandemic. For instance, research has shown that Hispanic populations in the USA also faced higher rates of COVID-19 infection and hospitalization compared to non-Hispanic white populations [26–28]. Additionally, Indigenous communities have experienced disproportionate impacts, including but not limited to the challenges related to unmet health needs [29]. Furthermore, South Asian communities in Ontario have encountered notable disparities, as evidenced by research illustrating heightened vulnerability and infection rates within this demographic [30].

Among White participants, the seroprevalence was < 4%, with a tendency for a higher rate among Black Canadians (11%). Seropositivity among Black persons was not statistically different from those among South Asian, East Asians and Hispanics, although it should be noted that the confidence intervals for the seropositivity rates among latter groups were wide due to the smaller sample sizes of these subgroups. The study acknowledges the smaller sample sizes of other groups, such as South Asians, East Asians and Hispanics and recognizes the need for further research to understand the seroprevalence rates among these subgroups. Additionally, by addressing the specific concerns and experiences of the Black community, the study can contribute to broader discussions on health disparities and equity that may benefit all populations.

Importantly, the seroprevalence rates detected in our study align with published information on the median household incomes of the communities studied [14–16]. This is consistent with the reported cases of COVID-19 based on income categories in Toronto, where individuals in lower-income categories were disproportionately affected by the virus compared to households with higher annual incomes [4]. These findings highlight the inequities in the impact of COVID-19 on different socioeconomic groups [4].

Our results suggest that persons less than 19 years of age were among those most likely to be seropositive. There is potential for younger individuals living in lower socioeconomic areas to contribute to the spread of COVID-19 in settings such as multigenerational homes. Higher seropositivity among persons less than 19 years has implications on strategies aimed at reducing the risk of COVID-19 among persons in school environments, including ways to enhance access to COVID-19 vaccines coupled with specially targeted educational measures, particularly within COVID-19 high-prevalence areas.

An accomplishment of the study is the successful enrollment of participants from the communities studied. Particularly within the Black community, there is often reluctance to participate in research studies due to various reasons, such as historical mistreatment and mistrust. However, the Community Advisory Group (CAG) recognized the importance of community engagement and trust building in overcoming these barriers. Through targeted outreach efforts and community partnerships, the study was able to establish a sense of trust and rapport with the Black community. This was crucial in encouraging individuals to participate and contribute to the study. The involvement of the CAG, which consisted of community leaders and representatives, played a vital role in bridging the gap between the researchers and the community. This facilitated oversampling of Black participants with proportionately more Black persons enrolled compared with the general population, as our results have indicated.

The successful enrollment from the communities studied not only allowed for a relatively high proportion of Black participants, but also ensured that the study findings were more relevant and meaningful to these communities. It also laid the foundation for future research collaborations and community-based interventions aimed at addressing health disparities and promoting health equity.

### Limitations

This report is limited because of the use of a convenience sample. It is likely that our ability to show differences between some ethno-racial groups was limited by the sample size. Some participants could have been exposed but had not yet seroconverted at the point of blood sampling. However, the effect of this potential bias would be to underestimate the rates of infection in the groups studied. It is possible that some persons who participated were more motivated to do so because of concerns regarding previous exposure to COVID-19. However, as shown in the results, this bias was likely present in about 14% of the study population. We did not examine some variables that might be of relevance, including but not limited to household density. The study covered a period of approximately 4 months during year 1 of the pandemic and the second wave of COVID-19; therefore, no conclusions can be drawn about subsequent waves of COVID-19.

One issue is the extent to which seroprevalence data might inform progress toward optimal community immunity [31–34]. It is possible that communities with higher levels of seropositivity (e.g. hot zones) could achieve herd immunity at lower levels of vaccine coverage than what is required in COVID-19 low incidence areas. This might be of relevance in some racialized communities with low vaccine uptake, where a lower percentage of vaccine coverage coupled with higher background seropositivity rates might be sufficient to achieve a high level of community immunity. This observation is not intended to inform policy, but rather to acknowledge the potential beneficial role of the combination of immunity from SARS-CoV-2 infection and vaccination [35].

## Conclusion

We have provided insights into the seroprevalence of SARS-CoV-2 infection among persons, including Black Canadians residing in COVID-19 high-prevalence zones in Ontario. This study examined the feasibility of a more extensive enrollment strategy within the Black community, while simultaneously providing insight into SARS-CoV-2 seroprevalence within a COVID-19 high-prevalence zone in Ontario. Overall, the study’s achievement in enrolling participants from the Black community demonstrates the feasibility of overcoming research participation barriers through community engagement and trust building. It serves as a stepping stone for future studies to continue fostering community partnerships and inclusivity in research. The sample size represents the largest report of SARS-CoV-2 seroprevalence among Black Canadians and one of few such reports in North America. We observed a tendency of greater seropositivity among individuals less than 19 years of age. There appears to be a tendency for Black Canadians who resided in a lower socioeconomic region (the northwest region of the Greater Toronto Area and Peel area) to more likely to be seropositive when compared with their White Canadian counterparts.

## Data Availability

The data and material are not available to the public.
